# WOMAC, Kujala Score, and Knee Injury and Osteoarthritis Outcome Score for Quality of Life Thresholds for Predicting Increased and Decreased Likelihood of Failure to Improve Quality of Life After Total Knee Replacement

**DOI:** 10.7759/cureus.69853

**Published:** 2024-09-21

**Authors:** Maximiliano Barahona, Macarena A Barahona, Camila Amstein

**Affiliations:** 1 Department of Orthopedics, Hospital Clinico Universidad De Chile, Santiago, CHL

**Keywords:** knee arthroplasty, knee osteoarthritis/ koa, patient-reported outcome measure, quality of life (qol), total knee joint replacement (tka)

## Abstract

Background

Improvement in quality of life is the primary goal following total knee arthroplasty (TKA). Patient-reported outcome measures (PROMs) have become the standard for evaluating TKA results, capturing the patient’s perspective. However, PROMs face challenges such as inconsistent presurgery data collection and ambiguity in determining clinical significance. Establishing reliable thresholds for success and failure is crucial for comparing outcomes.

Purpose

To determine cutoff values for the Western Ontario and McMaster Universities Osteoarthritis Index (WOMAC), Kujala score, and Knee Injury and Osteoarthritis Outcome Score for Quality of Life (KOOS-QL) that significantly change the likelihood of success (TIS) or failure (TIF) to improve self-reported quality of life one year after TKA compared to the baseline probability of the studied cohort.

Methods

A retrospective study was conducted to evaluate PROMs following conventional cruciate-retaining (CR) TKA without patellar replacement. Patients were evaluated during 2022 and 2023, with a minimum one-year follow-up. A total of 161 successful evaluations were identified, representing 81% of all CR TKA procedures without patellar replacement performed between January 2018 and June 2022 at a single university hospital. Assessments included the three dimensions of the WOMAC scale (pain, stiffness, and function), Kujala score, and KOOS-QL. The primary outcome was to determine the threshold value of each PROM that significantly reduced or increased the likelihood of “same or worse” self-perceived improvement in quality of life compared to the cohort. Logistic regression with 200 iterations was used for statistical analysis.

Results

The threshold for improvement success was <4 for WOMAC-Pain, <1 for WOMAC-Stiffness, <15 for WOMAC-Function, >70 for Kujala, and >62 for KOOS-QL. Meanwhile, the threshold for increased failure was >7 for WOMAC-Pain, >3 for WOMAC-Stiffness, >26 for WOMAC-Function, <55 for Kujala, and <41 for KOOS-QL.

Conclusions

The study successfully established significant thresholds for success and failure in improving quality of life following CR TKA without patellar replacement. The identified thresholds for WOMAC-Pain, WOMAC-Function, and Kujala scores have good-excellent discrimination and can be confidently used to estimate sample sizes and compare quality of life improvements post-TKA.

## Introduction

Satisfaction and improvement in the quality of life are the primary goals following total knee arthroplasty (TKA) [1]. Historically, the satisfaction rate has been reported at around 80%; however, recent findings suggest an increasing trend up to 90% [2], thus narrowing the gap with total hip arthroplasty [3].

Several methods for comparing TKA results have been proposed. Patient-reported outcome measures (PROMs) have become the standard over the last decade, aiming to capture the patient’s perspective [4]. Despite their utility, PROMs have limitations [5]. Challenges include inconsistent presurgery data collection and ambiguity in determining clinical significance when comparing mean or median scores across groups.

The most common thresholds for PROM-based assessments are the minimal clinically important difference (MCID) and the patient acceptable symptom state (PASS), which are complementary concepts [6]. MCID represents the threshold at which patients notice a significant change, while PASS is the 75th percentile of the accumulative distribution of successful cases [7]. However, establishing a reliable threshold for failure can be problematic, as the 75th percentile for unsuccessful cases is often not reported. This lack of data makes it difficult to compare treatments, particularly when considering non-inferiority studies.

To establish a threshold for success and failure, it is paramount to compare some types of outcomes using PROMs. For example, robotic-assisted techniques offer increased alignment precision and consistency, but clear benefits in PROMs have yet to be determined [8,9]. The author posits that many studies fail to detect differences due to reliance on mean or median PROM comparisons. A more appropriate method might be comparing the rate of success or failure based on qualitative thresholds of the PROM, given that consistency is one of the main benefits of robotic assistance TKA.

The aim of this study was to determine cutoff values for the Western Ontario and McMaster Universities Osteoarthritis Index (WOMAC), Kujala score, and Knee Injury and Osteoarthritis Outcome Score for Quality of Life (KOOS-QL) that significantly change the likelihood of success or failure to improve self-reported quality of life one year after TKA compared to the baseline probability of the studied cohort.

## Materials and methods

We conducted a retrospective study to evaluate PROMs following conventional cruciate-retaining (CR) TKA without patellar replacement, with a minimum one-year follow-up period between 2022 and 2023. In-person evaluations were performed by a physiotherapist with expertise in post-surgical rehabilitation and research-focused assessments on TKA at least one year after surgery. We identified 161 successful evaluations, representing 81% of all CR TKA procedures performed without patellar replacement between January 2018 and June 2022 at a single university hospital.

The assessments included PROMs measured using the three dimensions of the Western Ontario and McMaster University scale (WOMAC: pain, stiffness, and function), the Kujala score, and the KOOS QL. Additionally, satisfaction and self-perception of improvement in quality of life were evaluated using the Goodman scale [10]. The anchor question chosen for this study was, “How much did your knee surgery improve your quality of life?” This is the final question of the Goodman scale, which assesses self-perceived improvement in quality of life using a six-point Likert scale (Table 1). Responses of “1,” “2,” “3,” or “4” were categorized as “same or worse,” while “5” and “6” indicated a “much better” quality of life (Table 1).

**Table 1:** Anchor question used in this study The question is the last item on the scale published by Goodman et al., and it has six alternatives to the question, “How much did your knee surgery improve your quality of life?”

Response	Label	Categorization in this study
1	Worse	Same or worse
2	Same as before	
3	Low improvement	
4	Moderate improvement	
5	Great improvement	Much better
6	More than I ever dreamed	

The primary outcome was to determine the threshold value of each PROMs that significantly reduced and increased the likelihood of “same or worse” self-perceived improvement in quality of life compared to the cohort. Among the total cohort, 21 TKA patients (13.04%) perceived their quality of life as “same or worse” post-procedure, establishing a baseline probability of 0.130. The value that increased the probability of success was labeled threshold for increased success (TIS), and the value that increased the probability of failure was labeled threshold for increased failure (TIF).

For statistical analysis, first, a descriptive analysis of the accumulative frequency was performed. Then, a logistic regression model with 200 iterations was employed, using the anchor question as the dependent variable and each PROM as the independent variable. The probability and the corresponding 95% CI of achieving a “same or worse” outcome after TKA were calculated for each PROM value using the delta method. The threshold value was defined as the first value where the CI did not include the cohort’s overall probability of 0.130. Discrimination quality was assessed using the area under the receiver operating characteristic (ROC) curve, categorized following the Hosmer-Lemeshow proposal as <0.6 = low, 0.6-0.69 = discrete, 0.70-0.79 = moderate, 0.80-0.89 = good, and >0.9 = excellent [11]. Statistical analyses were conducted using Stata version 17.1.

## Results

A total of 161 TKAs were included in the analysis, with 92 (57.1%) being female. The median age was 67 years (range: 47-88; IQR: 62-70), and the median BMI was 31.2 (range: 21-44; IQR: 28.4-33.6).

**Figure 1:**
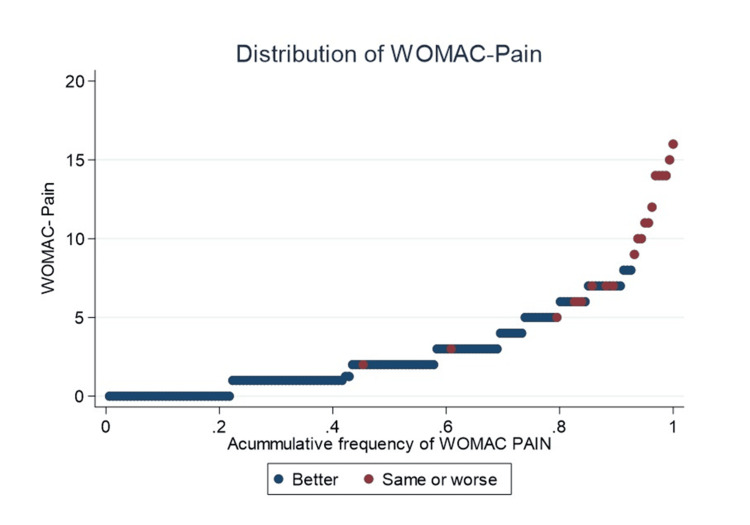
Accumulative frequency of WOMAC-Pain In red, patients who perceive their quality of life as “same or worse” compared to before the prosthesis are highlighted, whereas in blue, those who perceive their quality of life as significantly “better” are highlighted. WOMAC, Western Ontario and McMaster Universities Osteoarthritis Index

The cumulative distribution of each PROM was depicted in the figures. For WOMAC-Pain, 80% of the cohort scored 5 or less, with two TKAs (9.5%) reporting “same or worse” quality of life (Figure 1).

**Figure 2:**
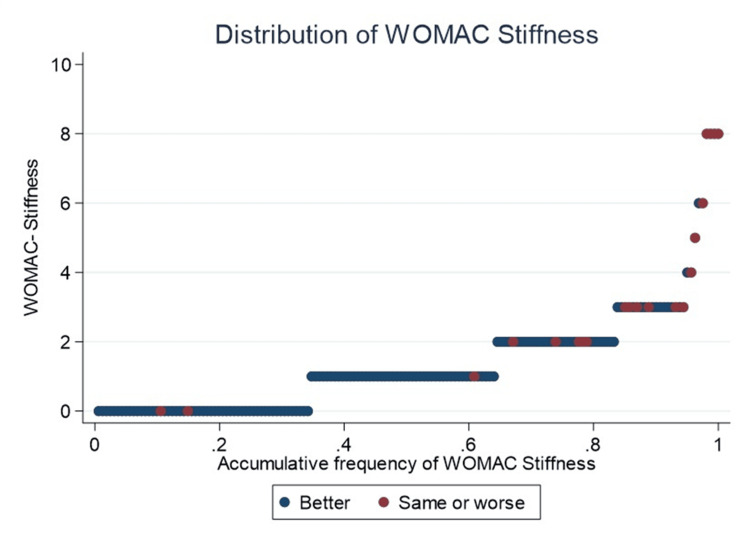
Accumulative frequency of WOMAC-Stiffness In red, patients who perceive their quality of life as “same or worse” compared to before the prosthesis are highlighted, whereas in blue, those who perceive their quality of life as significantly “better” are highlighted. WOMAC, Western Ontario and McMaster Universities Osteoarthritis Index

Regarding WOMAC stiffness, 65% of the cohort scored 1 or less, including three patients with undesirable outcomes (Figure 2).

**Figure 3:**
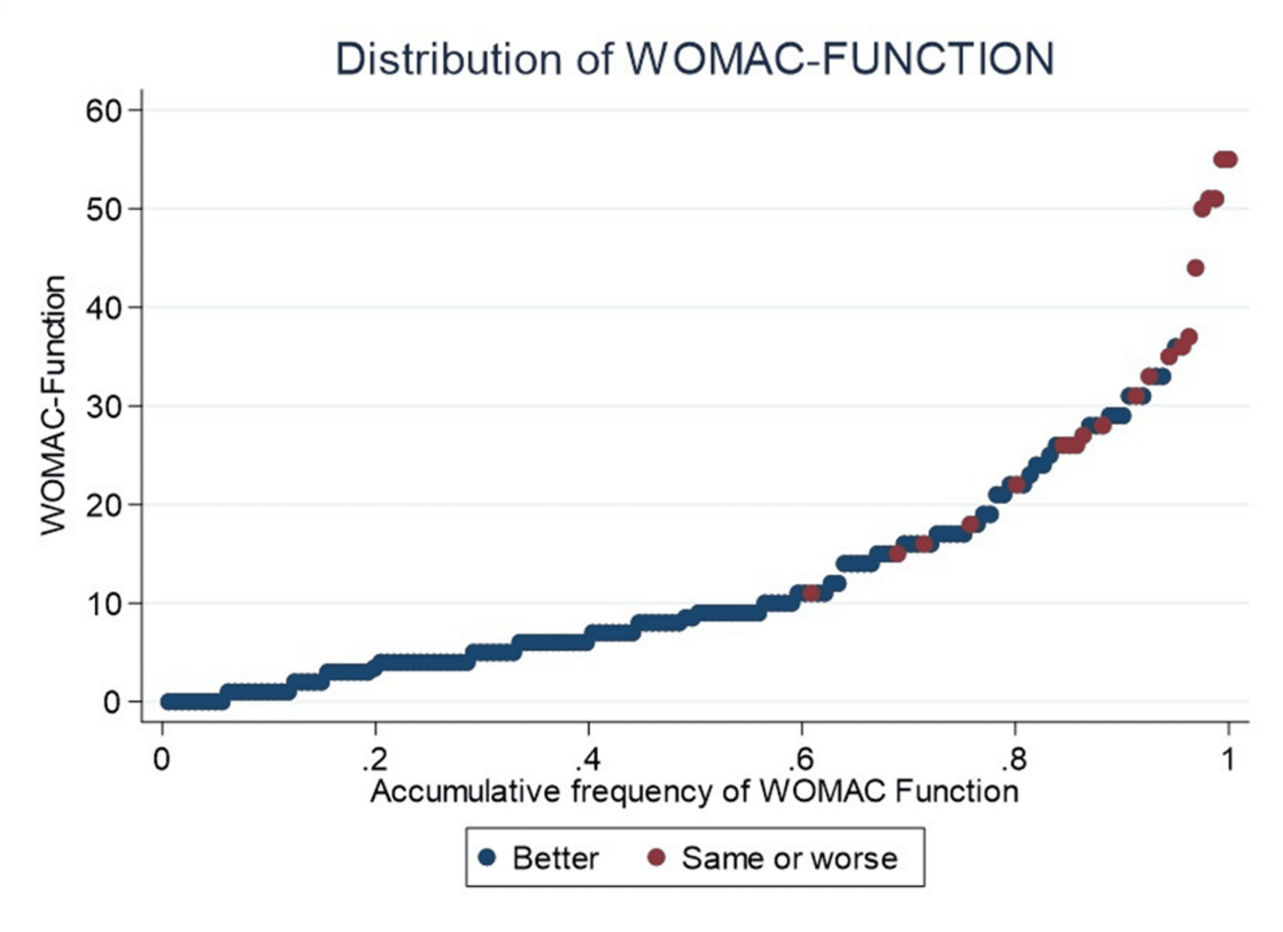
Accumulative frequency of WOMAC-Function In red, patients who perceive their quality of life as “same or worse” compared to before the prosthesis are highlighted, whereas in blue, those who perceive their quality of life as significantly “better” are highlighted. WOMAC, Western Ontario and McMaster Universities Osteoarthritis Index

Sixty percent of the cohort scored 10 or less on WOMAC-Function, all of whom belong to the “much better” group (Figure 3).

**Figure 4:**
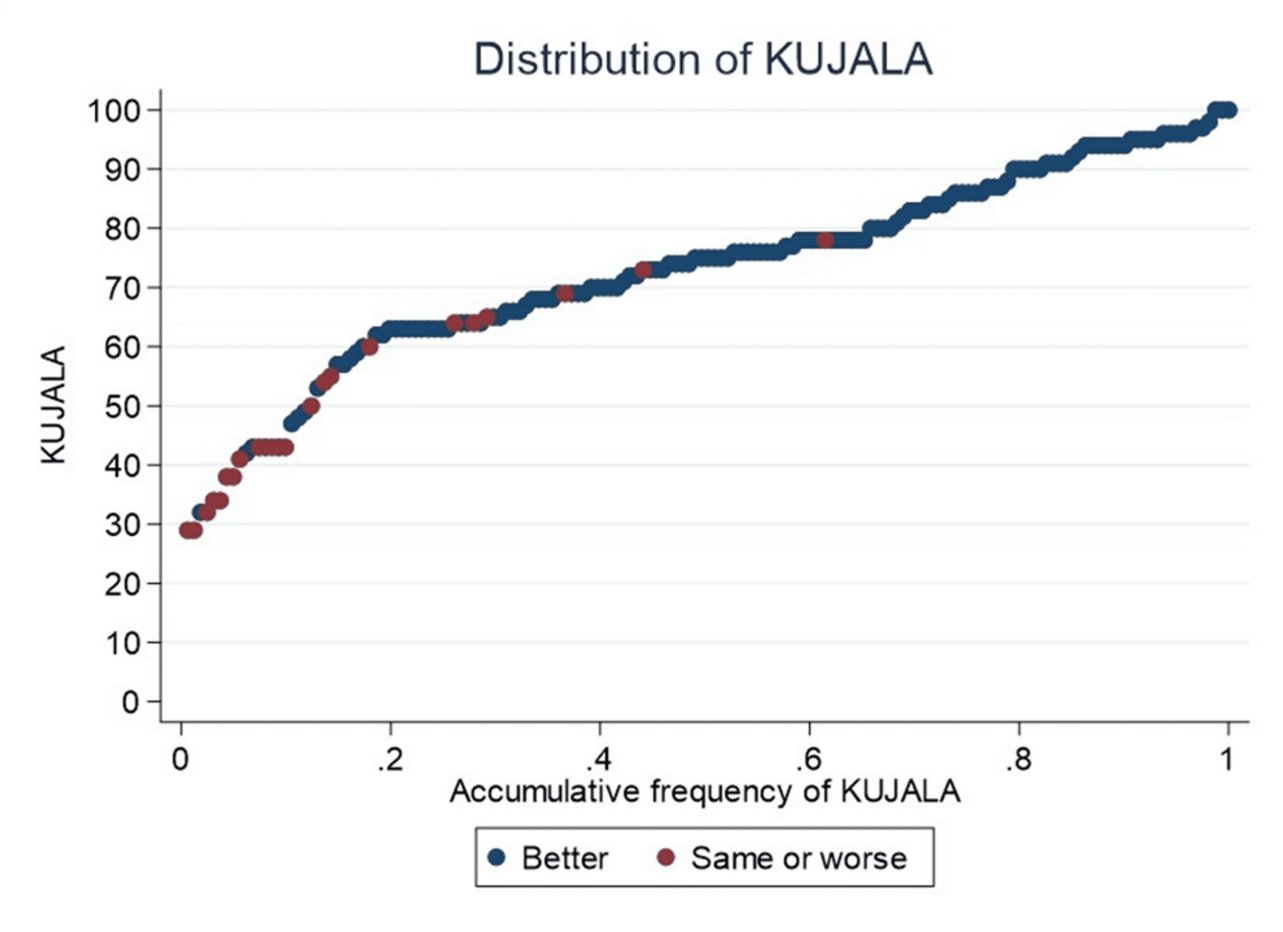
Accumulative frequency of Kujala In red, patients who perceive their quality of life as “same or worse” compared to before the prosthesis are highlighted, whereas in blue, those who perceive their quality of life as significantly “better” are highlighted.

On the Kujala scale, 60% of patients reported scores of 70 or higher, with only two (9.5%) reporting “same or worse” quality of life after TKA (Figure 4).

**Figure 5:**
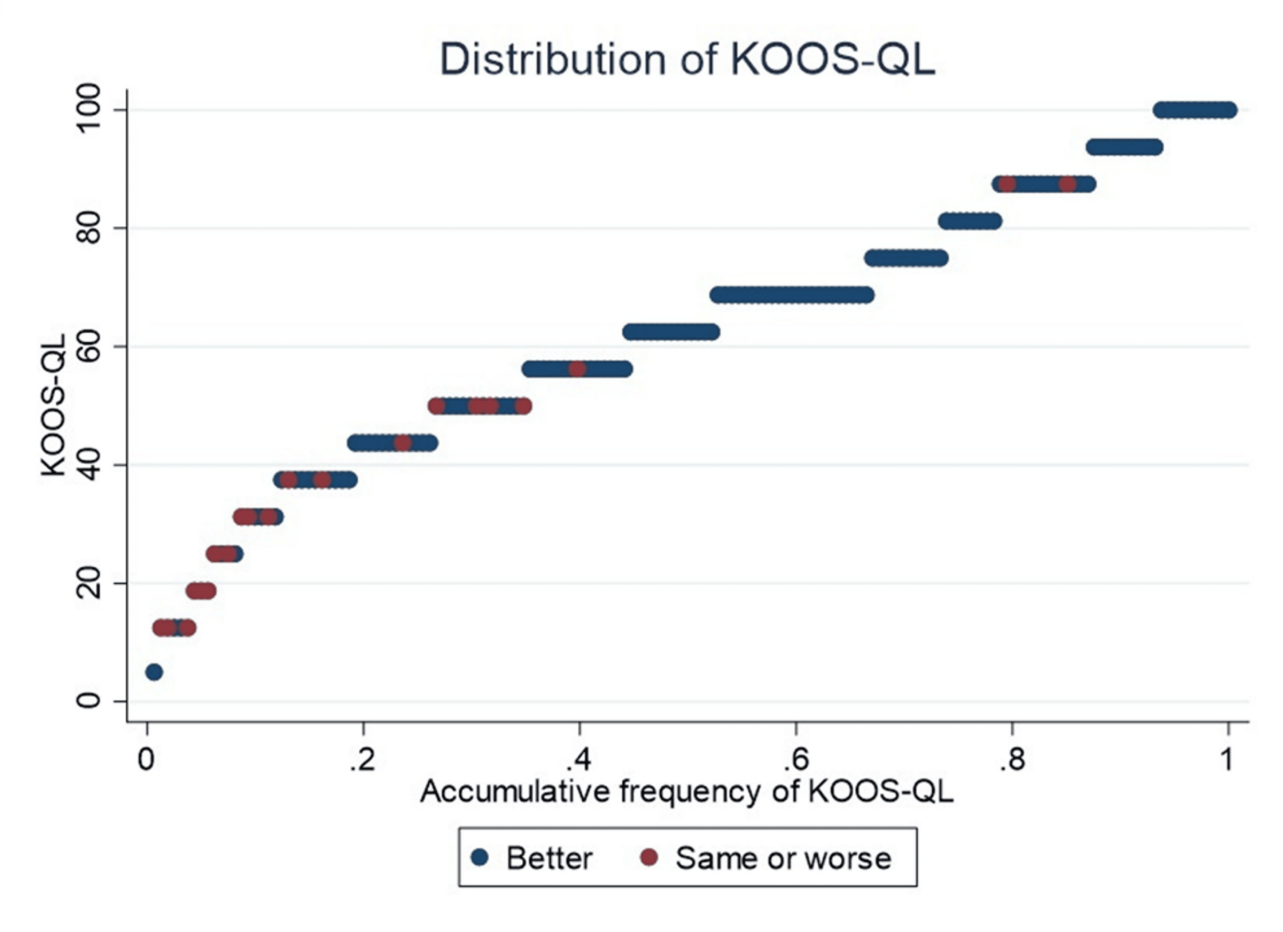
Accumulative frequency of KOOS-QL In red, patients who perceive their quality of life as “same or worse” compared to before the prosthesis are highlighted, whereas in blue, those who perceive their quality of life as significantly “better” are highlighted. KOOS-QL, Knee Injury and Osteoarthritis Outcome Score for Quality of Life

All TKAs reporting “same or worse” quality of life had KOOS-QL scores below 60, except for two cases (Figure 5).

The cutoff values were chosen after calculating the 95% CI using the delta method for each PROM value and observing that the probability of the cohort (p = 0.130) was not contained within that CI. The chosen values are summarized in Table 2 and their probability in Table 3.

**Table 2:** TIS and TIF likelihood for each PROM The area under the ROC curve and the respective 95% CI are shown in the last column. AUC ROC, area under the curve of the receiver operating characteristic curve; PROM, patient-reported outcome measures; QL, quality of life; TIF, threshold for increased failure; TIS, threshold for increased success; W, Western Ontario and McMaster Universities Osteoarthritis Index

PROM	TIS	TIF	AUC ROC
W-Pain	<4	>7	0.94 (0.89-0.99)
W-Stiffness	<1	>3	0.84 (0.73-0.94)
W-Function	<15	>26	0.93 (0.89-0.98)
Kujala	>70	<55	0.89 (0.82-0.96)
Koos-QL	>62	<41	0.83 (0.73-0.94)

**Table 3:** Probability estimated for the category “same or worse” of the variable self-perceived improvement of quality of life after TKA for each of the selected TIS and TIF compared to the baseline probability P, probability; TIF, threshold for increased failure; TIS, threshold for increased success; W, Western Ontario and McMaster Universities Osteoarthritis Index; TKA, total knee arthroplasty

PROM	Baseline P	P TIS	P TIF
W-Pain	0.13	0.064 (0-0.128)	0.359 (0.197-0.521)
W-Stiffness	0.13	0.060 (0.012-0.107)	0.286 (0.143-0.429)
W-Function	0.13	0.064 (0.011-0.117)	0.305 (0.150-0.4605)
Kujala	0.13	0.068 (0.011-0.125)	0.273 (0.136-0.409)
Koos-QL	0.13	0.072 (0.018-0.125)	0.223(0.136-0.311)

The best discrimination was achieved by WOMAC-Pain and WOMAC-Function, with ROC curve areas above 0.90, indicating excellent discrimination. Kujala achieved an area under the curve of 0.89, considered very good discrimination. WOMAC stiffness and KOOS-QL had lower discrimination, with the 95% CI dropping below 0.80, indicating only moderate discrimination (Table 2). The probability of reporting the “same or worse” category for the threshold of each PROM is summarized in Table 3.

## Discussion

The primary finding of this study is the identification of thresholds that increase or decrease the likelihood of success in improving quality of life after at least one year of CR TKA with patellar preservation. The importance of these cutoff points lies in the fact that they are calculated based on a question that compares the patient's self-perception of their current quality of life relative to their previous state. Furthermore, they can be used for sample calculations in non-inferiority (TIF) or superiority (TIS) studies and for comparing success or failure rates in experimental studies that aim to evaluate the consistency of the procedure rather than the procedure itself, such as the use of robotic assistance without changing the alignment philosophy.

Despite the increased use of PROMs, there is variability in the methods used to evaluate clinically significant success or failure and subsequent interpretation of results [12]. A previous study set the PASS for satisfaction with their current symptoms in W-pain at 5 (IC95%, 4-6) and for W-Function at 22 (IC95%, 20-24) in 510 patients, but it did not report the cumulative distribution of the not satisfied cohort [13]. The difference in the cutoff value could be explained as the PASS value being very sensible to the anchor question used [14]. In this study, the question includes a comparison with a previous status, while in Escobar et al., the question is for satisfaction with current symptoms [13]. No previous PASS for Kujala and KOOS-QL has been reported in the literature for TKA according to a previous systematic review [14], so it is not possible to compare the results of this study.

Regarding discrimination, WOMAC-Pain and WOMAC-Function demonstrated the best results, with areas under the ROC curve exceeding 0.90, indicating excellent discrimination. This aligns with patient expectations of reduced pain and improved function after TKA [15-17]. Kujala is a scale originally designed to evaluate patients with anterior knee pain due to patella-femoral problems [18]; nevertheless, it has been used to evaluate anterior knee pain in patients after TKA [19-21]. This report shows good discrimination for improvement in quality of life, which should encourage other researchers to include this PROM in their follow-up after TKA.

We consider that the methodology used in this study enhanced our results. Despite other strategies that have been described, we used the anchor method as it is recommended by the FDA to conduct this type of study [22]. Additionally, the chosen anchor question has the strength of being a validated question for TKA patient assessment, validated in different languages, and intended to evaluate their quality of life at least one year after surgery compared to their status before the surgery [10,23,24]. Moreover, TIS and TIF represent an increase in likelihood with respect to the whole cohort for success or failure, respectively, estimated using logistic regression analysis with the bootstrap technique, providing a robust statistical approach to determining these values [25].

However, the study has some limitations, primarily due to the sample size. To address this, we applied logistic regression with bootstrap techniques, which mitigates the impact of smaller samples through resampling. Despite this limitation, our study offers valuable insights into defining PROM thresholds after TKA.

Caution is advised when applying these thresholds to other types of TKA, such as posterior sacrifice or cases with patellar replacement; similarly to PASS, TIS and TIF should vary depending on the context used [14]. Further studies with larger sample sizes are recommended to validate these findings and ensure broader applicability.

## Conclusions

The present study has successfully established significant thresholds for success improvement and increased failure in patients undergoing cruciate retaining TKA without patellar replacement for WOMAC, Kujala, and KOOS-QL, utilizing as an anchor question the self-perceived improvement in quality of life. The identified values for TSI and TIF for WOMAC-Pain, WOMAC-Function, and Kujala had good discrimination and can be confidently utilized to estimate sample size and compare quality of life improvement post-TKA.
